# The New Theory of the Action of Chloroform

**Published:** 1890-09

**Authors:** Thomas Gaddes


### THE NEW THEORY OF THE ACTION OF CHLOROFORM.*

BY THOMAS GADDES, L.D.S., ENG. & ED.

It has been pointed out in the editorial pages of the Dental Journals, that an operation with "a small dose of chloroform is a Dangerous thing." That statement was based upon certain experiments by Dr. Lauder Brunton, the results of which appeared to be born out by the clinical experience of many observers, and by the evidence so frequently obtained at inquests, viz. :—that (by way of showing that death was not due to an incautious overdose) the patient was not fully anæsthetised, or was recovering from the more profound effects of chloroform, when, during the removal of the "last" tooth, or the insertion of the final stitch, death, ensued. In such cases it was argued that chloroform was a direct sedative to the action of the heart; that it reduced the excitability of the cardiac (motor) centres of the sympathetic; that, common sensation not being overcome, the "trivial" operation was perceived by the sensory centre in the medulla; that a resulting impulse was transmitted to the adjacent centre of the pneumogastric and, through that nerve, to the heart inhibiting its already impaired action, failure of the heart ensuing. Thus death from shock and syncope was held to take place; and it was further maintained that, had the patient been fully anæsthetised throughout the operation, shock would have been avoided and syncope, at least from that cause, averted. To day that teaching of the physiological action of chloroform has to be, not simply amended but almost entirely reversed. The experiments of the Second Hyderabad Commission give grounds for a different interpretation of the phenomena attending the administration of chloroform from what has hitherto generally been accepted. The results obtained by the First Commission were so much at variance with those acquired by other experimenters that a Second Commission was organized, having among its numbers that distinguished physician and scientist already referred to—Dr. T. Lauder Brunton, F.R.S. The Second Hyderabad Commission confirmed the conclusions arrived at by the preceeding one and, furthermore made several important discoveries, some 500 animals having been killed in the experiments. The report of the commission has not yet been published in a complete form, although important selections from it have at different times appeared in the *Lancet*, to which journal I am indebted for the information which I possess and for the extracts which follow.

These extracts do not by any means cover the whole ground traversed by the experiments. They are, however, sufficient to show that certain hypotheses as to the action and the safety or danger of chloroform are not in accord with the conclusions obtained from the recent and the most elaborate series of experiments ever carried out in that connection. The extracts are arranged in such order as to bear upon the successive stages in which death was supposed to occur in the case depicted above.

"The three series of experiments show that simple chloroform poisoning causes paralysis of the respiratory centre, and then gradual death, the heart being the last organ in the body to die. And, however concentrated the chloroform may be, it never causes a sudden death from stoppage of the heart."

"Chloroform, when given continuously by any means which insures its free dilution with air, causes a gradual fall in the mean blood-pressure, provided the animal's respiration is not impeded in any way, and it continues to breath quitely without struggling or involuntarily holding of the breath. But it must be clearly understood that such fall of blood-pressure is in no sense a danger, in any case which is fit for an operation, unless it is excessive—that is to say, unless an overdose of chloroform is inhaled.

"That struggling during chloroform inhalation, or anything which interfered with the breathing in any way, such as holding the breath or aspyxia, produced irregularities in the circulation and in the action of the heart.

"Chloroform has no power of increasing the tendency to either shock or syncope during operations. Every operation that ingenuity could suggest, or that has ever been supposed to be dangerous under chloroform, was performed by the Commission in every stage of Chloroformisation, without any effect upon the heart, the pulse, or the blood-pressure.

"That inhibition of the heart's action and slowing of the circulation with rapid fall of pressure, caused by irritation of the vagus, proved to be a safeguard—rather than a danger,—preventing the anæsthetic from being carried too rapidly from the lungs to the nerve centres. That an effect precisely similar to that caused by electrical stimulation of the vagus is produced through the same nerve: (*a*) in the holding of the breath, which occurs in the early stages of chloroform administration; (*b*) in asphyxia; and, (*c*), sometimes after the respiratory centre is paralysed in the later stages of chloroform poisoning."

As soon as this safeguard action of the vagus was demonstrated, it became clear "that chloroform and shock were not associates but incompatibles; and that the supposed capricious action of chloroform upon the heart was due either to the stimulating effect of concentrated vapor upon the nervous system (the vagus), or to the effect of the asphyxial blood upon the nerve centres, resulting in the exclusion of the poison from the system, and not the direct effect of the absorbed poison upon the heart or its nerves."

"Though fall of pressure is inseperable from chloroform administration, there is never the least danger if it falls regularly, and if the inhalation is stopped directly the state of the cornea shows that the patient is 'under.' Regularity of the blood-pressure depends entirely upon regularity of the respiration. Any irregularity, therefore, in the fall of blood-pressure during chloroform inhalation, indicates irregularity of, or interference with the respiration; and, *per contra*, any irregularity of, or interference with the respiration, at once causes irregularity in the fall of blood-pressure; but as long as the respiration is regular and not interfered with, the fall of the blood pressure will exactly correspond with it, and will cease long before a dangerous point is reached, *if the inhalation is stopped when the cornea becomes insensitive*, or other signs show that the patient is 'under.' If the respiration is kept up without struggling, holding the breath, or asphyxia, chloroform may be given slowly or quickly, freely and with perfect confidence, without the slightest risk to the patient.

The Commission has demonstrated that the aim of the surgeon must be to give chloroform so that the blood-pressure should fall regularly throughout the whole administration, and that the blood-pressure can only be kept free from irregularity of the breathing. The chloroform must therefore, be inhaled in such a way that the breathing is natural and regular throughout. Feeling the pulse during chloroform inhalation is no guide whatever either to the blood pressure or to the one thing necessary for safety, which is to keep it regular; and it has been shown above that the pulse is of no value as a sign of approaching danger, since it is only affected dangerously (*a*) when the respiration has been interfered with, or (*b*) by an over-dose. Lastly, in order to keep the breathing regular, the whole of the administrator's attention must be concentrated upon this point alone; and it is therefore clear that if, as is now recommended in most of the text books, part of the chloroformist's attention is to be given to the pulse, an important element of danger comes into the administration.

At present, being in the wilds of the Rocky Mountains and amidst scenes of the "far west," I have not any means of referring to the pages of the *Record*, or to other literature bearing upon the subject. But at the earliest moment I hasten to place before the readers of the Journal, with which I was so long and happily associated, this doctrine of the action of chloroform, which, if not entirely new, is different from that which was generally accepted, even by the highest authorities, and also enunciated in the pages of the *Dental Record.* If I remember rightly it was the received opinion in Scotland, as well as that of Sir J. Y. Simpson, Mr. Syme, Sir J. Lister, &c., that death results from failure of respiration and not from syncope, and it was, therefore, of the utmost importance to watch with the greatest care the respiration alone, discarding the pulse, the pupil, &c. This, too, is the lesson urged by the Second Hyderabad Commission.

The question of anæsthesia by chloroform in dental operations has been discussed by the Odontological Society, and some years ago the consensus of opinion was that, owing to the high percentage of fatalities, chloroform should only be administered in exceptionally severe cases (in which nitrous oxide or ether was not suitable), and then, by preference, at the patient's residence. Should the results and recommendations of the Second Hyderabad Commission be proved correct and satisfactory in experience, the data so gathered will necessitate the reconsideration of the queston by the dental profession.—*London Dental Record.*

---

### Communications.

### AMERICAN ACADEMY OF DENTAL SCIENCE.

*Editor of the American Journal of Dental Science:*

The Twenty Third annual meeting of the American Academy of Dental Science will be held in Boston, on Wednesday, November 12, 1890.

The annual address will be delivered by W. W. H. Thackston, M.D., D.D.S., of Farmville, Va.

EDWARD N. HARRIS, D.D.S.,
Cor. Secretary,
2 Park Square, Boston, Mass.

---

### THE DENTAL PROTECTIVE ASSOCIATION.

*Editor of the American Journal of Dental Science:*

